# Simultaneous LTE Signal Propagation Modelling and Base Station Positioning Based on Multiple Virtual Locations

**DOI:** 10.3390/s22155917

**Published:** 2022-08-08

**Authors:** Seong-Yun Cho

**Affiliations:** Department of Mechanical Automotive Engineering, Kyungil University, 50 Gamasil-gil, Hayang-eup, Gyeongsan-si 38428, Gyeongsangbuk-do, Korea; sycho@kiu.kr

**Keywords:** base station positioning, LTE signal propagation model, reference signal received power (RSRP) measurements, multiple virtual locations (VLs), sum of the squared residuals (SSR)

## Abstract

In the Long Term Evolution (LTE) system, the Signal Propagation Model (SPM) and the location information of the base stations are required for positioning a smartphone. To this end, this paper proposes a technique for estimating the SPM and the location of the base station at the same time using location-based Reference Signal Received Power (RSRP) information acquired in a limited area. In the proposed technique, multiple Virtual Locations (VLs) for a base station are set within the service area. Signal propagation modelling is performed based on the assumptions that a base station is in each VL and the RSRP measurements are obtained from the corresponding base station. The residuals between the outputs of the estimated SPM and the RSRP measurements are then calculated. The VL with the minimum sum of the squared residuals is determined as the location of the base station. At the same time, the SPM estimated based on the corresponding VL is selected as the SPM of the base station. As a result of the experiment in Seoul, it was confirmed that the positions of seven base stations were estimated with an average accuracy of 40.2 m.

## 1. Introduction

Location information of a pedestrian is recognized as being very important for emergency rescue and location-based services and has been estimated and provided based on various methodologies [1]. To this end, Global Positioning System (GPS)/Global Navigation Satellite System (GNSS) is the most common method in outdoor environments [2], with mobile/wireless network-based wireless location technology also used in various ways [3,4,5,6]. However, GPS/GNSS can normally only be used outdoors and wireless network-based positioning is generally only available indoors or in outdoor areas where infrastructure is installed. On the other hand, wireless positioning based on a mobile communication network is most suitable when considering the availability of positioning in all indoor and outdoor spaces. Wireless positioning techniques include Cell-ID, time of arrival [7,8], time-difference of arrival [9], fingerprinting [10,11], etc. Among them, a fingerprinting or a trilateration technique can be used for positioning using signal strength information [12]. In order to construct an efficient database in the fingerprinting technique or to implement a trilateration technique, a Signal Propagation Model (SPM) and location information of the base stations are basically required in the Long Term Evolution (LTE) system [13,14]. This information can be also used to create a fingerprint database. However, this information is managed as the property of the mobile carrier and is not provided free of charge to positioning technology developers or location-based service providers. This is the first motivation of this study.

The power of the signal transmitted from the antenna of each base station is attenuated as it propagates through space. In addition, the power is more attenuated as the signal penetrates structures such as buildings. In urban areas, therefore, SPMs are geographically different due to different spatial structures. Because of this, analyzing the Reference Signal Received Power (RSRP) measured at several locations at the same distance from a base station reveals that the signal variance is very large. Therefore, to develop LTE signal-based positioning technology, different SPMs must be used at different base stations [15,16]. This is the second motivation of this study.

Considering this situation, the following problem exists: the location information of the base station is required for Signal Propagation Modelling (SPMing) but is not provided by the mobile carrier. Here, SPMing means regressing the coefficients of a SPM. Even if the RSRP measurements can be converted into distance information using the SPM provided in the literature, it is impossible to accurately estimate the location of the base station based on the existing trilateration method because of the large variance of the RSRP measurements [17]. This is the third motivation of this study.

With these motivations, in this paper, a technique that can simultaneously perform SPMing and base station positioning is proposed. From now on, this technique is called Simultaneous SPMing and base station Positioning (SMaP). First, multiple Virtual Locations (VLs) for base stations are created in the service area. The VLs can be easily created in a grid structure akin to building a fingerprint database within the service area. Based on the assumption that there is a base station in each VL, SPMing is performed using the location-based RSRP measurements. The residuals between the output of the estimated SPM and the RSRP measurements are then calculated. One VL with the minimum Sum of the Squared Residuals (SSR) value can be extracted. This VL is determined as the location of the base station. In addition, the SPM estimated based on the VL determined as the location of the base station is selected as the SPM suitable for the surrounding area of the corresponding base station.

The SPMs estimated based on the VLs far from the actual location of the base station do not properly reflect the attenuation characteristics of RSRP over distance. In this case, the calculated SSR values are large. Therefore, it is determined that the hypothesis that the VL with the minimum SSR value is the closest to the actual location of the base station among the multiple VLs is appropriate, and this hypothesis is to be verified through the experimental results.

Experiments were performed to verify the performance of the proposed technique. The RSRP measurements with GPS/GNSS-based location information are collected on the road through a vehicle equipped with a signal acquisition device, Nemo Handy. Among the collected signals, the proposed technique is applied after extracting the signal from Korea Telecom (KT), a mobile communication company. The actual location information of base stations in the service area was supported by KT for research purposes. Experimental analysis shows that the proposed technique can simultaneously perform SPMing and base station positioning. It also shows that the hypothesis that the VL with the minimum SSR value is closest to the actual location of the base station is true.

The rest of this paper is organized as follows. The characteristics of the RSRP measurements are described in Section 2, and SMaP is explained in Section 3. In Section 4, experimental analysis is conducted and, finally, Section 5 concludes this paper.

## 2. Characteristics of LTE RSRP Measurements

In this section, the characteristics of LTE RSRP measurements in urban areas are analyzed based on the published literature and the limitation of the path loss model expressed based on the SPM for wireless location is identified. Based on this, the limitation of ranging using RSRP measurements is described and it is also explained that positioning using the converted ranging data is impossible in the LTE system.

### 2.1. Analysis of Signal Characteristics Based on the Published Literature

As the signal transmitted from the antenna propagates through the air, the power of the signal is attenuated, and this path loss is usually modelled as a log-distance model. The most famous path loss models are the *α*-*β*-*γ* model and the close-in model [18]. The parameters used in these models have different values depending on the environment, and various studies have been conducted to estimate these values in the line-of-sight environment and the non-line-of-sight environment [19,20].

In the wireless location domain, since the measurement is signal strength, the received power model is used more than the path loss model. The received power model can be expressed as follows [21,22,23].

(1) P(r, s, t)=P(r)+Ψ(s)+a(t),

where *P*(*r*) is the strength of the signal received according to the transmission distance *r*, and Ψ(*s*) and *a*(*t*) are random variables that are affected by the location of the terminal *s* and time *t*, respectively.

In (1), Ψ(*s*) is called long-term (or slow) fading, and *a*(*t*) is called short-term (or fast) fading [21,22]. Ψ(*s*) is generated by multipath signals from buildings and other obstacles, is also called shadowing, and is modelled as a lognormal distribution. However, it is known that this effect cannot be eliminated [23]. *a*(*t*) can be removed by averaging received signal strength over time [24]. However, it is difficult to completely eliminate this effect.

In the LTE system, the short-term fading included in multiple measurements acquired at one location can be removed by averaging RSRP data received over a certain period. However, the long-term fading is a component that appears depending on location and changes according to circumstances, so it is not easy to model and remove this effect. Therefore, the following simplified model is widely used in the positioning area because it is not possible to take into account unknown parameters in the real environment [17,25,26,27].

(2) $$P(r)=P(r_{R})-10\alpha \cdot \log_{10}(r/r_{R})+w(\sigma ),$$

where *α* is the average path loss index, and *P*(*r*) and $P(r_{R})$ are RSRP data obtained at distance *r* and $r_{R}$ from the base station, respectively. $r_{R}$ is a reference distance and is generally set to a distance close to the base station, such as 1 m. That is, $P(r_{R})$ is a parameter related to the transmission power of a signal.

**Figure 1:**
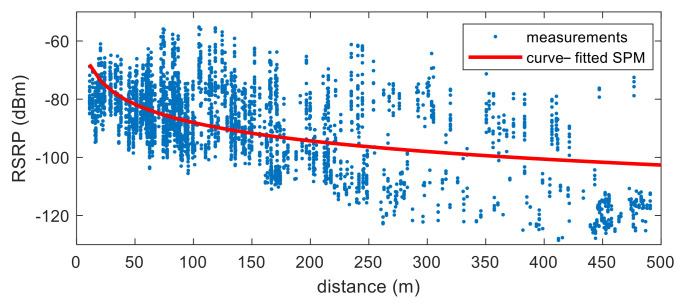
RSRP measurements and results of SPMing performed based on (2).

**Figure 2:**
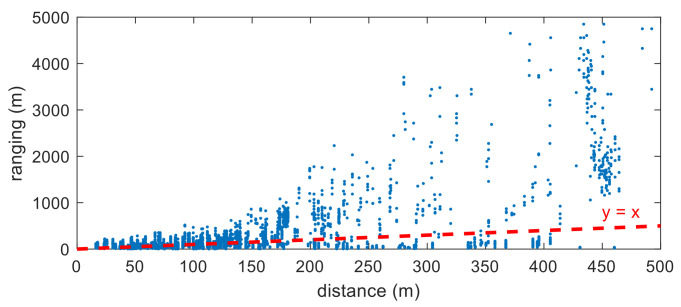
Ranging information converted from RSRP measurements in Figure 1.

Additional power attenuation caused by buildings, long-term fading, is always a phenomenon in real environments. However, this is region specific and cannot be expressed as a generalized value. Unfortunately, this effect is assumed to be contained in *w* in (2), and the resulting phenomenon is analyzed in Figure 1 and Figure 2. Figure 1 shows the RSRP measurements according to distance and the results of SPMing performed based on (2). As can be seen in this figure, the standard deviation is very large when RSRP data obtained from an urban area is modelled as (2). Eventually, this phenomenon appears as a characteristic of the signal when performing LTE signal-based positioning in an urban environment with many multipath signals.

### 2.2. Limitation of LTE RSRP-Based Positioning

When *n* RSRP measurements are obtained, (2) can be expressed as following matrix form [17]:

(3) $$Y=H{\left[\begin{array}{cc} P(r_{R}) & \alpha \end{array}\right]}^{T}+{\left[\begin{array}{ccc} w_{1} & \cdots & w_{n} \end{array}\right]}^{T},$$

where

(4) $$Y={\left[\begin{array}{ccc} P(r_{1}) & \cdots & P(r_{n}) \end{array}\right]}^{T},\;\mathrm{and}$$

(5) $$H=\left[\begin{array}{cc} 1 & -10\cdot \log_{10}({\bar{r}}_{1}/r_{R})\\ \vdots & \vdots \\ 1 & -10\cdot \log_{10}({\bar{r}}_{n}/r_{R}) \end{array}\right],$$

where ${\bar{r}}_{i}$ is calculated as the distance between the actual location of the base station and the location where the *i*^th^ RSRP measurement is obtained.

$P(r_{R})$ and *α* can be estimated using the least squares method as follows [17]:

(6) $$\left[\begin{array}{c} \hat{P}(r_{R})\\ \hat{\alpha } \end{array}\right]={\left(H^{T}H\right)}^{-1}H^{T}Y.$$

After estimating the coefficients of the SPM, (2) can be expressed as an equation for distance as follows [28]:

(7) $$r=r_{R}\cdot 10^{(\hat{P}(r_{R})-P(r))/10\hat{\alpha }}.$$

This is the basic formula for RSRP-based ranging. Figure 2 shows the ranging information converted from the RSRP measurements shown in Figure 1. In this figure, the horizontal axis is the distance between the base station and the location where the RSRP measurement is obtained, and the vertical axis is the value obtained by converted RSRP information into distance. What is expected is that ranging information is plotted around the graph of *y* = *x*. However, it can be seen from this figure that the ranging errors are very large, and the variance of the errors tends to increase gradually with distance.

In addition, if the exact location of the base station is not known, $P(r_{R})$ and *α* cannot be estimated exactly, resulting in larger ranging errors. That is, to estimate the location of the base station, the values of these two parameters are necessary, and to calculate the parameter values, the location of the base station is necessary. Therefore, it can be seen that the two estimates have an interdependent relationship. Moreover, if the location of the base station is estimated by a general trilateration method using the converted ranging information, it can be confirmed through experiments that it does not converge to a constant value due to large ranging errors. That is, when the error of the initial position is estimated by the iterative least squares method, it is diverged. Therefore, this is the limitation of LTE RSRP-based positioning. That provides the major motivation for the methodology of this paper.

In summary, it is impossible to estimate the position of the base station using the conventional RSRP-based distance conversion information and the trilateration method due to the large variance of the RSRP measurements in the LTE system. In order to estimate the location of the base station while avoiding the limitations of the LTE RSRP-based positioning, a VL-based SMaP technique is proposed in the next section.

## 3. Simultaneous SPMing and Base Station Positioning

**Figure 3:**
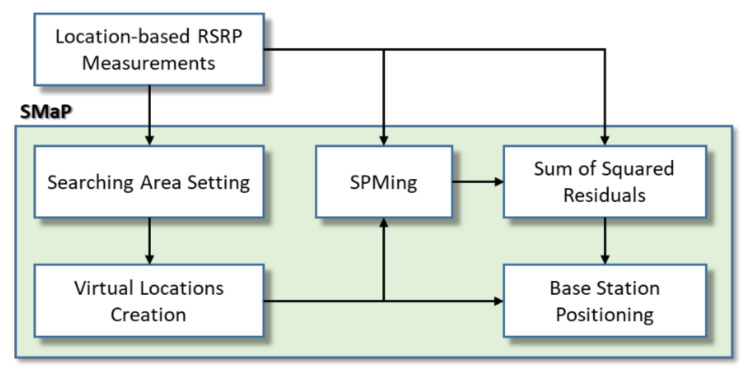
Functional blocks and procedure of SMaP.

In order to perform SMaP, functions and procedures are described in Figure 3. First, location-based RSRP measurements are obtained through a signal measurement device and SMaP is performed based on this. Based on Figure 3, the idea of this paper to perform SMaP is described sequentially.

**Figure 4:**
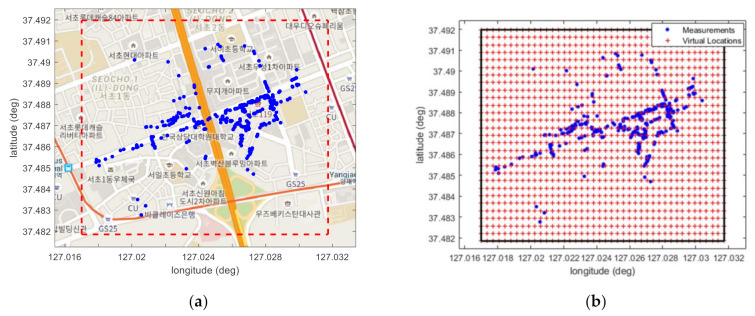
An example of RSRP measurements and VLs. (**a**) Locations of RSRP measurements and the searching area; (**b**) grid points for VLs.

Step−A Searching Area Setting: Figure 4a shows the location information of signals acquired from the KT base station of Physical Cell Identity (PCI)−144 in Seocho−dong, Seoul, Korea. The locations at which the measurements were obtained are satellite-based augmentation system-based differential GPS information. Based on these measurements, a searching area is set for the positioning of the base station. In this figure, the blue dots are the locations of the RSRP obtained through a vehicle test, and it can be seen that measurements were obtained only in limited areas such as roads. The red dashed square shows the searching area.

Step−B Virtual Locations Creation: VLs of a base station in the searching area are generated in the form of grid points,

(8) $$VL^{j}={\left[\begin{array}{cc} Lat^{j} & Lon^{j} \end{array}\right]}^{T},\;\;\;j\in \{1,\;2,\;\cdots ,\;N_{VL}\},$$

where $\left(Lat^{j},\;\;Lon^{j}\right)$ is the latitude and longitude coordinates of the *j*^th^ VL ($VL^{j}$) and $N_{VL}$ is the number of VLs.

In the searching area, the VLs of the base station are created in the form of grid points. In this study, a total of 1218 VLs were created by setting the interval of the grid points to 40 m, as shown in Figure 4b. The interval of the grid points was determined experimentally, and detailed explanations are given through the experimental results in the next section.

Equation (3) can be rewritten as follows:

(9) $$\left[\begin{array}{c} P(r_{1}^{j})\\ \vdots \\ P(r_{s}^{j}) \end{array}\right]=\left[\begin{array}{cc} 1 & -10\cdot \log_{10}({\bar{r}}_{1}^{j}/r_{R})\\ \vdots & \vdots \\ 1 & -10\cdot \log_{10}({\bar{r}}_{s}^{j}/r_{R}) \end{array}\right]\left[\begin{array}{c} P{\left(r_{R}\right)}^{j}\\ \alpha^{j} \end{array}\right]+\left[\begin{array}{c} w_{1}^{j}\\ \vdots \\ w_{s}^{j} \end{array}\right],$$

where

(10) $$r_{i}^{j}=\sqrt{{\left((Lat^{j}-Lat_{i})R_{Lat}\right)}^{2}+{\left((Lon^{j}-Lon_{i})R_{Lon}\cos Lat^{j}\right)}^{2}},$$

where *s* is the total number of RSRP measurements and the superscript *j* is the number of each VL. $\left(Lat^{j},\;\;Lon^{j}\right)$ and $\left(Lat_{i},\;\;Lon_{i}\right)$ are the latitude and longitude coordinates of the *j*^th^ VL and the *i*^th^ RSRP measurement acquisition location, respectively. $R_{Lat}$ and $R_{Lon}$ are the Earth radius in the latitude and longitude directions, respectively, and can be calculated as [29]:

(11) $$R_{Lat}=\frac{R_{0}(1-e^{2})}{{\left(1-e^{2}\sin^{2}Lat\right)}^{3/2}},\;\;\;R_{Lon}=\frac{R_{0}}{{\left(1-e^{2}\sin^{2}Lat\right)}^{1/2}},$$

where $R_{0}=6,278,137$ m and e=0.0818191908426 are the equatorial radius and eccentricity of the Earth’s ellipsoid, respectively.

Step−C SPMing: SPMing is performed based on the created VLs. Assuming that the base station is at each virtual location, the two coefficients $P{\left(r_{R}\right)}^{j}$ and $\alpha^{j}$ in (9) are estimated by the least squares method as in (6). This process is repeatedly performed based on all VLs, and the estimated coefficients are stored as follows: $\hat{P}{\left(r_{R}\right)}^{j}$ and ${\hat{\alpha }}^{j}$, where $j\in \{1,\;2,\;\cdots ,\;N_{VL}\}$.

Step−D Calculate the Sum of Squared Residuals: When this process is completed, the SSR for each VL is calculated as follows:

(12) $$SSR^{j}={\sum_{i=1}^{n}\left\{r_{R}\cdot 10^{(\hat{P}{\left(r_{R}\right)}^{j}-P(r_{i}^{j}))/10{\hat{\alpha }}^{j}}-r_{i}^{j}\right\}}^{2},$$

where $r_{i}^{j}$ is calculated as (10).

Step−E Minimum SSR-based Base Station Positioning: Finally, the position of the base station is determined as follows:

(13) $$P\hat{o}s=VL^{\lambda },$$

where

(14) $$\lambda =\underset{j\in \{1,\;2,\;\cdots ,\;N_{VL}\}}{\arg\;\;\min}(SSR^{j}).$$

It is the hypothesis of this paper that the VL with the minimum SSR value is closest to the base station among all VLs. Therefore, it is the main idea of this paper to estimate the VL with the minimum SSR value as the location of the base station according to this hypothesis. Proof of this hypothesis is carried out through experimental analysis in the next Section.

## 4. Experimental Analysis

**Figure 5:**
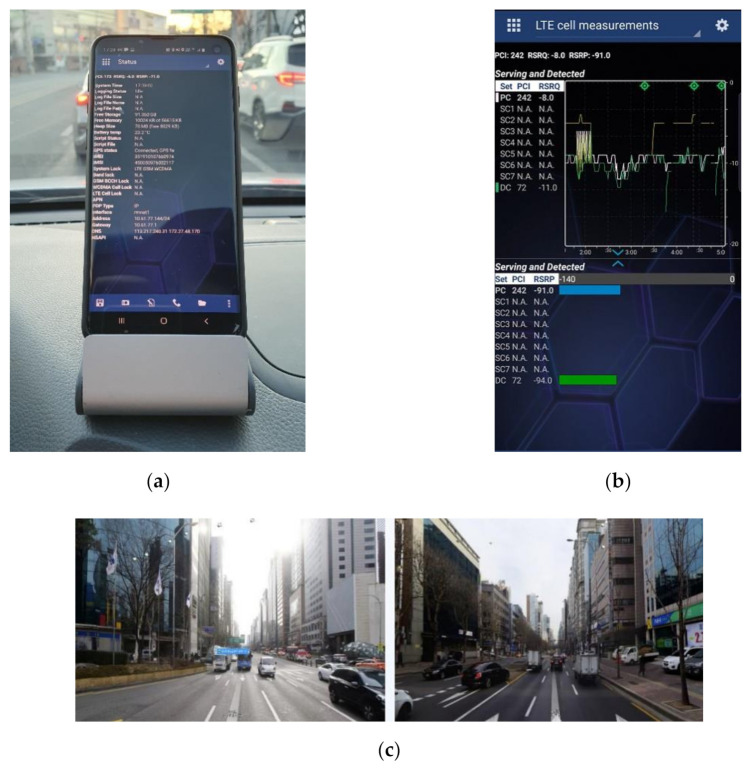
Measurement equipment and experimental environment. (**a**) LTE signal acquisition equipment; (**b**) an example of signal information obtained from Nemo Handy; (**c**) test environment viewed from the vehicle during the signal acquisition experiment.

Experiments were conducted to verify the performance of the proposed SMaP. As shown in Figure 4, the test site is the center of Seoul, Korea, and the target mobile carrier is KT. As shown in Figure 5a, a smartphone S10 equipped with Nemo Handy, an LTE signal acquisition app from Keysight Technologies, was mounted in one vehicle and data was collected while driving for about 3 h on the road in the service area. This data includes downlink information of the LTE system and DGPS-based location information from which the information is obtained. An example of the signal acquired from Nemo Handy is shown in Figure 5b. This figure shows the number of the serving PCI and RSRP information of the received signals, and the information was saved as a file with the extension nmf. Figure 5c shows the urban environment seen from the vehicle during the signal acquisition experiment. From this figure, it can be seen that the signal propagation environment is not good because the tall buildings are attached to each other in the urban area, but signal propagation is easy in the road direction.

Among the collected data, only the data corresponding to Band−8 (downlink center frequency is 954.3 MHz and bandwidth is 10 MHz) was extracted through parsing the saved file. After extracting each piece of RSRP information and time-synchronized GPS/GNSS-based location information together, SMaP was performed.

**Figure 6:**
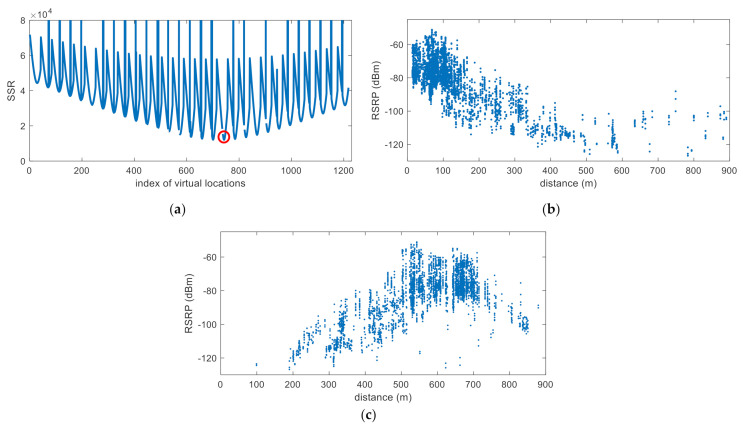
SSR and distance–RSRP information according to VL. (**a**) SSR according to VL; (**b**) distance–RSRP information reconstructed based on the VL with the minimum SSR value; (**c**) distance-RSRP information reconstructed based on an arbitrary VL.

**Figure 7:**
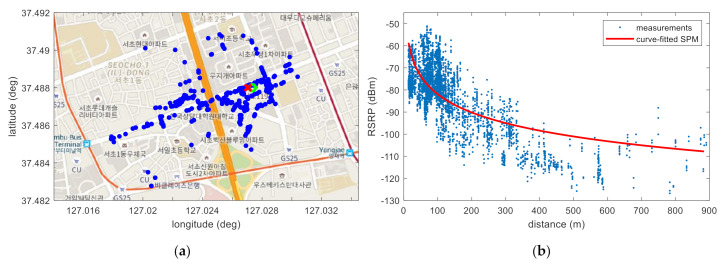

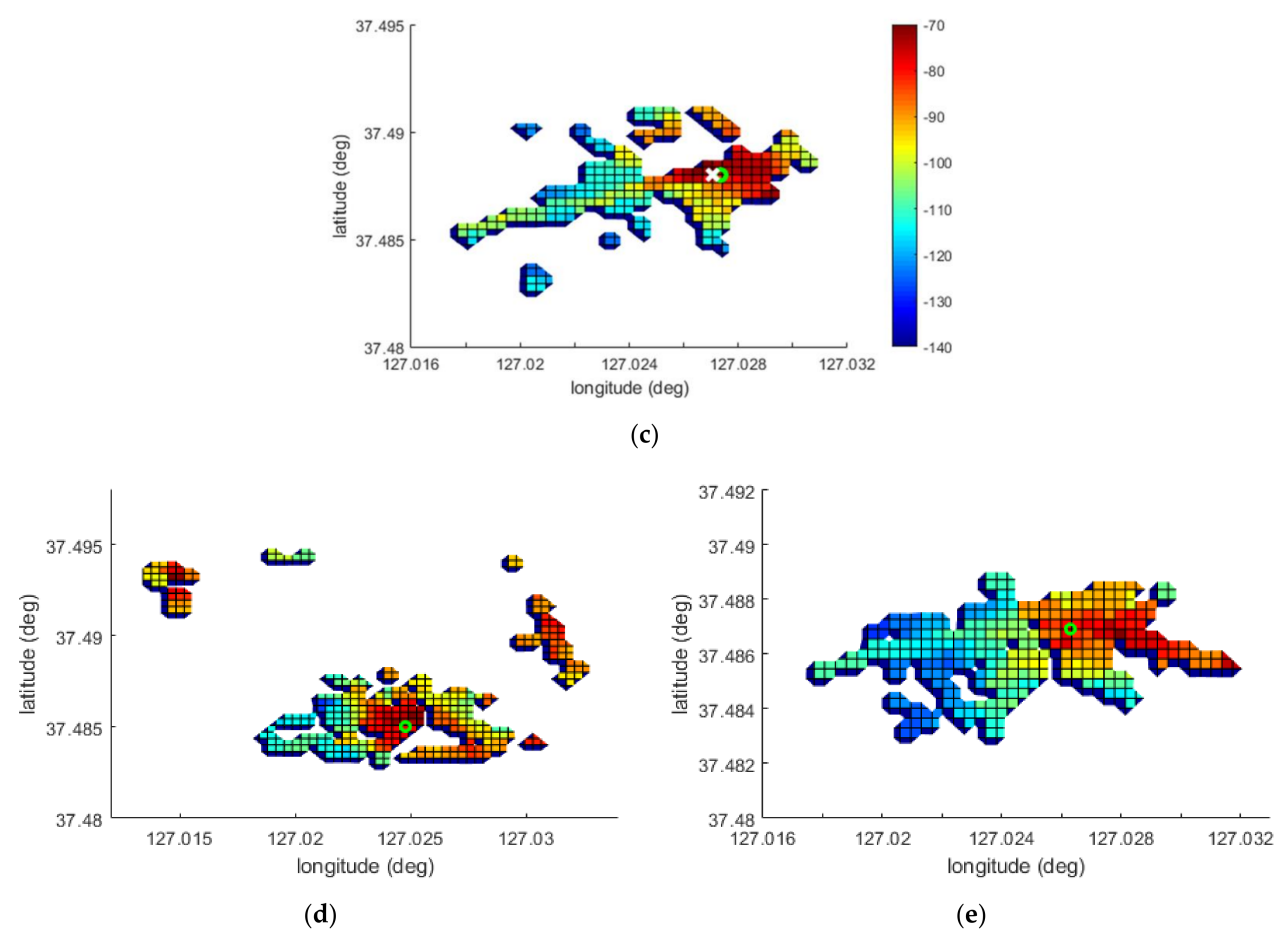
Results of SMaP and the comparison technique. (**a**) Result of positioning for the PCI–144 base station; (**b**) result of SPMing; (**c**) generated fingerprint database; (**d**) fingerprint database created in case of PCI–186 with repeaters; (**e**) fingerprint database created in case of PCI–176.

**Figure 8:**
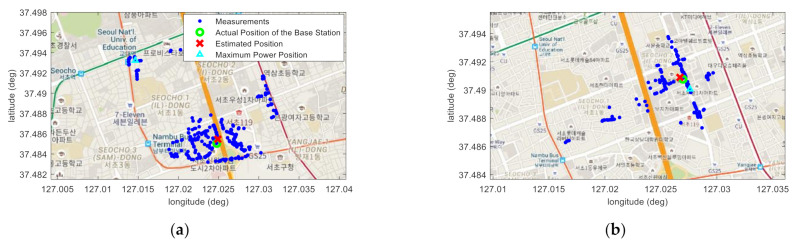

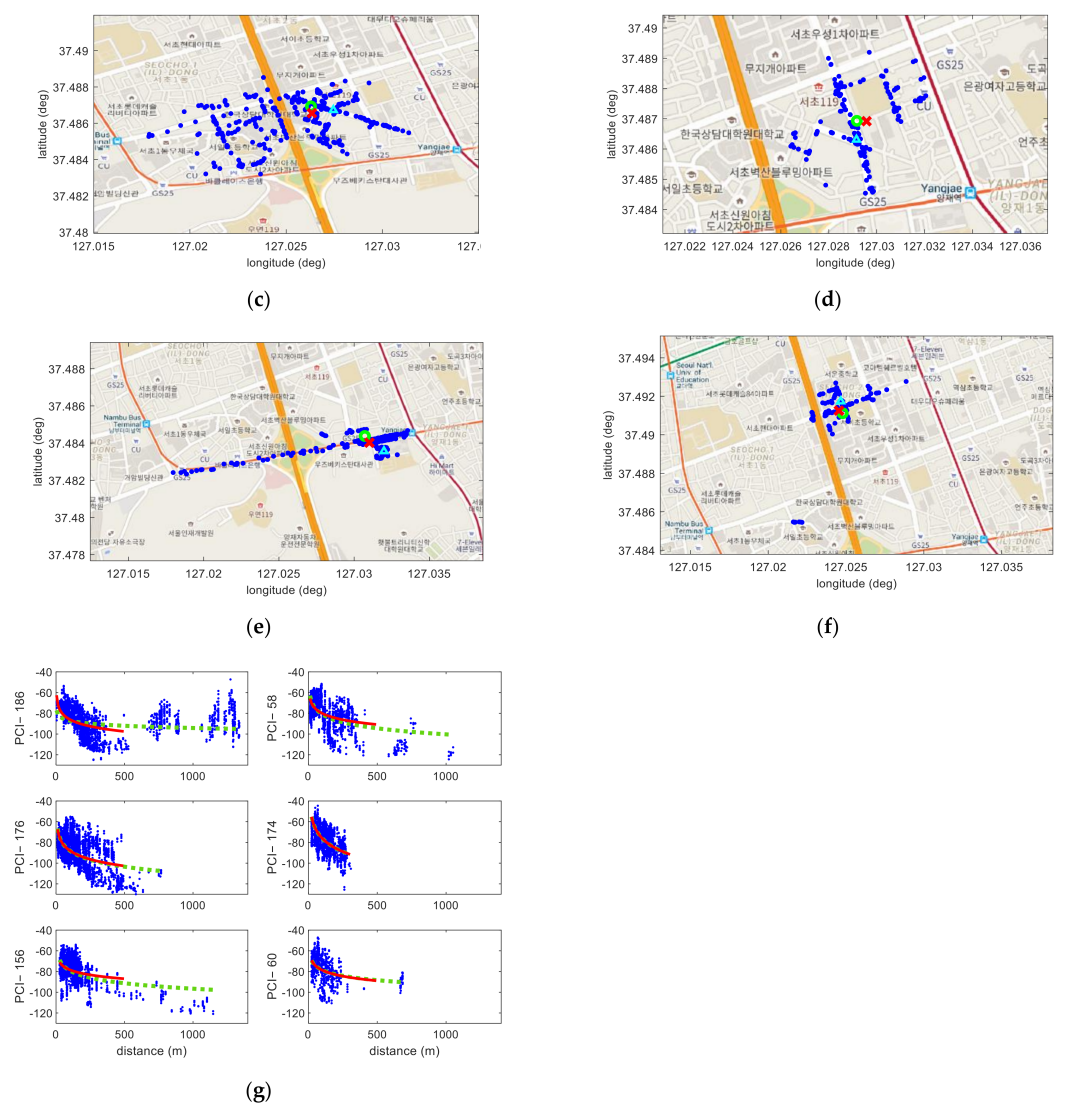
Results of base station positioning and SPMing. (**a**) PCI–186; (**b**) PCI–58; (**c**) PCI–176; (**d**) PCI–174; (**e**) PCI–156; (**f**) PCI–60; (**g**) SPMing.

Experimental analysis is performed in the following order. First, Figure 6 shows that the minimum SSR value according to the VL has adequate regularity to prove the hypothesis that the VL with the minimum SSR value presented in this paper is close to the true location of the base station. In this figure, it is also shown that only the result of SPMing performed on the VL with the minimum SSR value is regressed in the form of (2). By showing in Figure 7 that the estimated location of the base station is near the true location as a result of the SMaP performed based on this data, it is confirmed that the hypothesis presented in this paper is highly likely to be correct. Another hypothesis other than this one can be considered. That is, the position having the highest power among the acquired RSRP information may be estimated as the position of the base station. However, if there is a repeater, Figure 7d shows that this method cannot accurately estimate the location of the base station. By showing in Figure 8 the results of SMaP performed based on 6 PCI data different from the PCI shown in Figure 7, it can be sure that the hypothesis presented in this paper is correct. As a result of SMaP, it can be seen in Figure 8g that not only the location of the base station but also the coefficients of the SPM can be accurately estimated. A detailed description of each figure is as follows.

Figure 6 shows the signal processing of SMaP based on the measurements of the PCI–144 base station, shown in Figure 4, among the extracted data. The algorithm was run on 1218 VLs with a total of 4127 measurements. SPMing is performed after assuming that the base station is in each VL. The algorithm processing time took 9.7 s in Matlab.

Figure 6a shows the SSR according to the VL. From this figure, it can be seen that SSR is calculated as a different value for each VL and there is one VL with the minimum SSR value. Figure 6b shows the reconstructed distance–RSRP information based on this VL. Here, the distance was calculated through (10). From this figure, it can be confirmed that the RSRP measurements according to distance are distributed in the form of a log function. Figure 6c shows the distance–RSRP information reconstructed by assuming an arbitrary VL, other than the VL with the minimum SSR value, as the base station. From these figures, if there is an error in the location information of the base station, it can be inferred that a large error will occur in SPMing due to the incorrect distance–RSRP measurement information formed based on this error. Because of this, the calculated SSR has a large value. Therefore, it can be confirmed that it is appropriate to determine the VL with the minimum SSR value obtained through Figure 6a as the location of the base station.

Figure 7 shows the results of performing SMaP and the comparison technique. In Figure 7a, the green circle is the actual location of the base station and the red x is the estimated location. The solid line in Figure 7b is the estimates of RSRP according to distance based on the SPM estimated using the measurements. Measurements obtained at a distance of more than 500 m from the base station are mostly signals propagated through roads. The power of these signals is greater than the signals propagating toward the building, not the road. Sometimes, signals from a repeater are received in this area, as can be seen in Figure 7d. The signal provided by the repeater is the same as that provided by the base station, and the PCI information included in the signal is the same as the neighboring base station PCI. Therefore, this signal is mistaken as a signal transmitted from a base station rather than a repeater, and the RSRP information of the signal received at a long distance from the base station has a large value, which adversely affects SPMing. Therefore, only signals acquired within 500 m are used for SPMing.

Figure 7a shows the result of the base station positioning based on the SSR denoted in Figure 6a. Among all the VLs, the SSR calculated at one location has the smallest value, and this location was selected as the location of the corresponding base station. The estimation error was calculated as 31.52 m. That is, it can be confirmed that the location of the base station is accurately estimated because an error occurs within the interval of the grid points. In addition, Figure 7c shows the fingerprint DB constructed at 40 m intervals based on the measurements. In this figure, it can be seen that a larger RSRP is being measured near the base station. However, it is not possible to determine the location with the strongest RSRP as the location of the base station because it may not be possible to obtain the measurements at the exact location of the base station by vehicle collection.

Additionally, Figure 7d,e supports this explanation. This figure shows the fingerprint DB constructed based on the measurements of two different base stations. In the case of PCI–186, it seems that there are several base stations with the same PCI based on the signal strength information alone. This is because there are repeaters in certain buildings. As a result, it is possible to obtain a measurement with a large RSRP value even at a location far from the actual base station. In the case of PCI–176, it can be seen that the base station is near the place where the RSRP value is large. Since the base station is installed on the building, there are many multipath signals in the vicinity. As a result, the largest RSRP measurement is taken near the base station, not the location of the base station. For these reasons, determining the location with the largest RSRP measurement as the location of the base station can lead to large errors.

Figure 8 shows the results of base station positioning and SPMing of 6 base stations, and Table 1 and Table 2 summarize the experimental results. First, it can be seen from Table 1 that the positioning accuracy of base stations varies according to the interval of grid points. However, it is not possible to know the correlation of accuracy according to the interval. The reason is that the error characteristic of the signal of the LTE system varies depending on the surrounding environment, and the surrounding environment of each base station is different. Through this experiment, in this paper, the interval of the grid points is set to 40 m. In Figure 8a–f, the blue dots show the locations where the measurements were obtained, the green circle shows the true location of the base station, and the red x shows the base station location estimated through SMaP when the interval of the grid points is set to 40 m.

Figure 8a is the case where there are several repeaters, as shown in Figure 7d, and Figure 8c is the case where the measurements are spread widely around the base station, as shown in Figure 8b. In the rest, since the base station is installed near the road, the measurements are long distributed along the road and are distributed within a measurable distance toward the side of the road. In all cases, it can be confirmed that the locations of the base stations were accurately estimated through the SMaP proposed in this paper.

What makes this possible is in the SPMing shown in Figure 8g. The dotted line is the result of SPMing using all the measurements, and the solid line is that using only the measurements within 500 m from the VL. In the cases of PCI–186, 58, and 156, if SPMing is performed using both the signals transmitted from the repeaters and the signals propagated along the road, it is impossible to estimate the SPM that properly reflects characteristics of the signal propagation around the base station. However, by using only the measurements within a certain coverage, it is possible to estimate the SPM that relatively accurately reflects the signal propagation characteristics around the base station. The SPMing accuracy and the positioning accuracy of the base station have a close relationship. That is, when the SPMing is correct, the position of the base station can be accurately determined, and when the position of the base station is correct, the accurate SPM can be obtained. However, since the interval of the VLs is 40 m, it does not greatly affect base station positioning unless the SPMing is significantly wrong, such as with PCI–186.

The position indicated by a triangle in Figure 8a–f is the location with the maximum power among the measurements and was estimated as the location of the base station. The estimation errors are shown in Table 2. In the case of PCI–186, the problem is that the power of the repeater signal is the largest among the measurements, resulting in a very large positioning error. Other than that, the positions were estimated near the base stations. However, as described above, the base stations were installed on buildings or on power poles, and the measurements were acquired on the road. Thus, the signal with the maximum power among the acquired signals cannot be obtained right in front of the base station. In addition, signal synthesis by multipath signals occurs frequently in urban areas. Therefore, the positioning errors are relatively large.

Based on this result, it can be confirmed that the proposed method can accurately estimate the location of the base stations and perform SPMing at the same time only with measurements obtained in a limited area in an urban area. When SMaP is completed in this way, it is possible to establish the location information of all base stations in the service area and the SPM for the corresponding base station signals. This information can be used to generate data for various services. For example, it is possible to fill the gaps in the fingerprint DB. As shown in Figure 7c, if the DB is constructed with only measurements, there may be places where there is no data even around the base station. The information on these places can be filled in by estimation using the location information and SPM of the base station estimated based on the SMaP proposed in this paper. Through this, it can be expected that the performance of fingerprinting-based smartphone positioning can be improved [17].

## 5. Conclusions

In this paper, a technique that simultaneously performs base station positioning and propagation modelling of signals transmitted from the base station using location-based RSRP information obtained in a limited area, such as roads, was proposed. A search area was set based on the measurement locations and VLs in the form of grid points were set in the search area. SPMing was performed after assuming that the base station was located in each VL. The SSR was calculated for each VL. The VL with the minimum SSR value was determined as the location of the base station, and the result of SPMing performed based on this VL was determined as the SPM for this base station. Through experiments conducted in Seoul, Korea, it was found that the location of seven base stations were estimated with an average of 40.2 m accuracy. Based on this, it can be concluded that the three problems that motivated this study were solved. That is, SMaP estimates the locations of the base stations and at the same time estimates a different SPM for each base station. The positioning technique used here is a novel technique different from the existing RSRP-based positioning techniques and its accuracy and effectiveness have been confirmed. In addition, it can be expected that the proposed technique can easily and accurately generate base station location information and an SPM for each channel, which is basic information for LTE signal-based positioning. Through this, practical applications, such as estimating the location of a terminal for LTE system-based emergency rescue, can be applied. In the future, based on the information estimated through the results of this study, research automation technology for establishing the fingerprint DB for emergency rescue positioning will be conducted.

## Figures and Tables

**Table 1:** Summary of positioning errors according to interval of grid points.

	Interval	30 m	35 m	40 m	45 m	50 m
PCI						
144		42.42 m	42.42 m	31.52 m	28.34 m	72.66 m
186		47.81 m	37.62 m	49.67 m	48.70 m	30.61 m
58		33.50 m	0.89 m	32.71 m	29.52 m	20.15 m
176		43.87 m	31.62 m	41.51 m	26.16 m	71.36 m
174		27.46 m	34.89 m	44.52 m	47.65 m	40.83 m
156		67.84 m	74.59 m	51.20 m	75.97 m	41.61 m
60		43.83 m	30.15 m	30.28 m	12.65 m	30.28 m
Mean		43.82 m	36.03 m	40.20 m	38.43 m	43.93 m
Standard Deviation		12.71 m	21.68 m	8.76 m	20.80 m	20.50 m

**Table 2:** Summary of positioning errors according to positioning methods.

	PCI	SMaP	Maximum Power
Method			
144		31.52 m	84.37 m
186		49.67 m	1426.64 m
58		32.71 m	103.88 m
176		41.51 m	128.12 m
174		44.52 m	68.66 m
156		51.20 m	165.09 m
60		30.28 m	59.36 m
Mean		40.20 m	292.30 m
Standard Deviation		8.76 m	501.38 m

## Data Availability

Detailed data supporting the reported results can be found in all figures and tables in this paper.

## Abbreviations

The main abbreviations used in this paper are defined as follows.

PCI (Physical Cell Identity): ID set by each carrier to distinguish base stations in LTE system.

RSRP (Reference Signal Received Power): a type of LTE signal strength information received from the terminal. This information is used as the reception sensitivity for communication and is also used to estimate the location of the terminal in the positioning system.

SMaP (Simultaneous SPMing and base station Positioning): a technology that simultaneously performs SPMing and location estimation of a base station using the acquired location-based RSRP data.

SPM (Signal Propagation Model): pass loss model. The signal transmitted from the antenna of the base station gradually weakens as it propagates through the air. SPM is a model that expresses the attenuation phenomenon of RSRP information according to distance.

SPMing (Signal Propagation Modelling): process for regressing an SPM using the acquired location-based RSRP data.

SSR (Sum of the Squared Residuals): the sum of the squared values of the residuals. Here, the residual is the difference between the RSRP information calculated based on the SPM and the RSRP information measured at the same location. The SPM is the product of the SPMing performed assuming that there is a base station in one VL.

VLs (Virtual Locations): virtual locations of a base station created in the service area.
